# The effect of exercise self-efficacy on basic psychological needs in flight cadets: the chain mediating role of psychological resilience and perceived social support

**DOI:** 10.3389/fpsyg.2025.1701055

**Published:** 2026-01-14

**Authors:** Hongni Wang, Maojun Gong, Mingyu Liao

**Affiliations:** 1 School of physical education and health, Linyi University, Linyi, China; 2 Linyi Vocational College, Linyi, China; 3 Aviation Safety and Security College, Civil Aviation Flight University of China, Deyang, China

**Keywords:** basic psychological needs, exercise self-efficacy, flight cadets, perceived social support, psychological resilience

## Abstract

**Objectives:**

Based on social cognitive theory and self-determination theory, this study explores the mechanism by which exercise self-efficacy influences the satisfaction of basic psychological needs in flight cadets, focusing on the mediating roles of psychological resilience and perceived social support.

**Methods:**

A cross-sectional design was employed to conduct a questionnaire survey among 524 flight cadets (first-year: 301; second-year: 223; mean age: 19.2 ± 1.3 years) from the Civil Aviation Flight University of China. Exercise self-efficacy, psychological resilience, perceived social support, and basic psychological needs were assessed using validated scales. Path analysis and bootstrap analysis were employed to test the hypothesized chain mediation model.

**Results:**

Results indicated that exercise self-efficacy significantly positively predicted basic psychological needs (*β* = 0.157) and operated through three indirect pathways: (1) the mediating role of psychological resilience (Effect = 0.167); (2) the mediating role of perceived social support (Effect = 0.062); and (3) the chain mediating role of psychological resilience and perceived social support (Effect = 0.052). The total effect size was 0.439.

**Conclusion:**

Exercise self-efficacy is a crucial factor promoting basic psychological need satisfaction among flight cadets. The findings reveal a chain mediation mechanism where exercise beliefs enhance psychological resilience, which in turn facilitates social support perception, ultimately satisfying basic needs. Interventions aimed at boosting exercise self-efficacy and resilience are recommended to enhance the mental health of flight cadets.

## 1 Introduction

Flight cadets represent a unique population who not only face complex professional skill learning requirements but also endure stringent elimination-based selection pressures, placing extremely high demands on their psychological state. Aviation psychology research indicates that throughout the entire developmental process from cadet stage to professional pilot, individuals must cope with unique challenges including cognitive load management, high-pressure decision-making, and emotional regulation, while maintaining psychological acuity and emotional stability, as mental health status directly relates to flight safety (Yu et al., 2022). From a psychophysiological perspective, these psychological constructs are not merely cognitive states but serve as essential regulators of physiological arousal and stress responses. Research suggests that high levels of self-efficacy can buffer against maladaptive physiological reactivity (e.g., excessive heart rate or cortisol spikes) during high-intensity flight tasks, thereby justifying the examination of these variables within the domain of performance adaptation (Potgieter and Steyn, 2010). During flight training, cadets frequently encounter multiple stressors including technical mastery, psychological pressure, and assessment evaluations, which may affect the satisfaction of their basic psychological needs and subsequently impact training effectiveness and career development (Chen and Wu, 2022). Exercise self-efficacy, as an important psychological resource for individuals to cope with these challenges, is considered one of the key variables affecting the mental health and adaptive capacity of flight cadets.

Exercise self-efficacy refers to an individual’s belief in their ability to successfully complete specific exercise tasks, originating from social cognitive theory (Shengyao et al., 2024). Exercise self-efficacy is not only associated with individual exercise performance but may also positively impact mental health. For flight cadets, higher exercise self-efficacy means they have greater confidence in completing tasks when facing physical fitness tests and training challenges, potentially helping them better cope with the physical and mental stressors in flight training. Self-determination theory posits that individual mental health is closely related to the satisfaction of three basic psychological needs: autonomy, competence, and relatedness (Tang et al., 2020). Previous research has found associations between physical exercise and the satisfaction of these three basic psychological needs, which may subsequently affect individual mental health levels and enhance overall psychological well-being (Teixeira et al., 2012; Kermavnar et al., 2024). Exercise self-efficacy, as an important foundation for individual autonomous decision-making, may enable cadets to demonstrate stronger capabilities and skills in physical fitness tests, thereby enhancing their sense of belonging and connection within the pilot community (Goodyke et al., 2022). However, existing research still presents some controversy regarding the relationship mechanisms between exercise self-efficacy and basic psychological need satisfaction. Some studies emphasize direct associations between the two, while others propose the existence of mediating variables. This theoretical divergence indicates the need for further exploration of the relationship pathways between them, particularly their manifestation patterns in the special population of flight cadets. Based on existing theoretical foundations and empirical evidence, this study proposes.

*Hypothesis 1:* Exercise self-efficacy can positively influence the satisfaction of basic psychological needs among flight cadets, namely enhancing their autonomy needs, competence needs, and relatedness needs.

Psychological resilience, as an individual’s effective coping ability when facing setbacks and stress (Palamarchuk and Vaillancourt, 2021), may hold significant importance in the high-pressure population of flight cadets. Psychological resilience may not only be a core component of flight cadets’ psychological qualities but also a key factor determining whether they can successfully pass stringent selection processes (Tornero-Aguilera et al., 2024). Research indicates that higher exercise self-efficacy helps enhance individual psychological resilience, enabling them to demonstrate stronger resistance and recovery capabilities when facing challenges (Li et al., 2024). For flight cadets, the enhancement of exercise self-efficacy may indirectly affect the satisfaction of basic psychological needs through its association with psychological resilience. When flight cadets achieve success and progress in physical training, their exercise self-efficacy may improve, potentially correlating with enhanced psychological resilience, enabling them to better cope with various challenges and stressors in flight training, ultimately promoting the satisfaction of basic psychological needs. Therefore, this study proposes Hypothesis 2.

*Hypothesis 2:* Psychological resilience mediates the relationship between exercise self-efficacy and basic psychological needs among flight cadets.

Perceived social support refers to the degree of care, help, and support that individuals perceive from their external environment. Existing research indicates (Rutter, 2012) that social support includes emotional support (such as care and understanding) and instrumental support (such as practical help), which may help individuals better engage in physical exercise. For flight cadets, who face selection pressures and training difficulties, social support from instructors, peers, and family members may be particularly important. Previous research suggests that perceived social support may be associated with enhanced individual psychological resilience, helping them better cope with stress and setbacks (Dong et al., 2024). Meanwhile, adequate social support may directly satisfy flight cadets’ relatedness needs and satisfy their autonomy and competence needs by providing autonomous choices and ability affirmation. However, the specific manifestation patterns of these associations in the flight cadet population still require further validation. Therefore, this study proposes Hypothesis 3.

*Hypothesis 3:* Perceived social support mediates the relationship between psychological resilience and basic psychological needs among flight cadets.

Previous research provides empirical support for the chain pathway “exercise self-efficacy → psychological resilience → perceived social support → basic psychological need satisfaction.” Peng et al. (2025) found in their study of 1,613 Chinese adolescents that physical exercise significantly influenced self-efficacy through the mediating role of psychological resilience, validating the positive relationships among exercise, psychological resilience, and self-efficacy. In military academy cadet research, multiple studies have confirmed the associations between psychological resilience training and improvements in self-efficacy and coping abilities, indicating that psychological resilience and self-efficacy may be closely associated in high-pressure environments (Crane et al., 2019; Navickienė and Vasiliauskas, 2024). Furthermore, Yang et al. (2025) found through their research that personality traits can influence college students’ exercise behavior through the chain mediation effects of exercise self-efficacy and exercise motivation, providing empirical foundations for similar chain pathways. Despite the accumulation of research on these variables individually, several critical gaps remain. First, existing studies have primarily validated the partial links within our proposed model (e.g., exercise to self-efficacy, or resilience to social support) in isolation, rather than examining the complete sequential chain pathway. Second, most previous findings are based on general student populations or athletes. Flight cadets, as a unique group facing high-stakes selection pressure and rigorous military-style management, may exhibit distinct psychological mechanisms that current theories have not yet fully explained. Specifically, whether exercise self-efficacy can activate the chain reaction of “resilience-social support” to satisfy their basic needs remains empirically unverified. This study aims to bridge this gap by constructing a chain mediation model to explicitly test these pathways within the specific context of aviation education. Based on the above analysis and previous research evidence, this study further hypothesizes that exercise self-efficacy may influence flight cadets’ basic psychological needs through a chain pathway, namely exercise self-efficacy → psychological resilience → perceived social support → basic psychological needs. When flight cadets possess higher exercise self-efficacy, they may have greater confidence in physical training, which may be associated with enhanced psychological resilience, enabling them to better cope with the stresses and challenges of flight training. Stronger psychological resilience may make flight cadets more likely to obtain and perceive social support from instructors, peers, and family members, and this support may ultimately satisfy their basic psychological needs. Therefore, this study proposes Hypothesis 4.

*Hypothesis 4:* Exercise self-efficacy influences flight cadets’ basic psychological needs through the chain mediation of psychological resilience and perceived social support.

**Figure 1:**
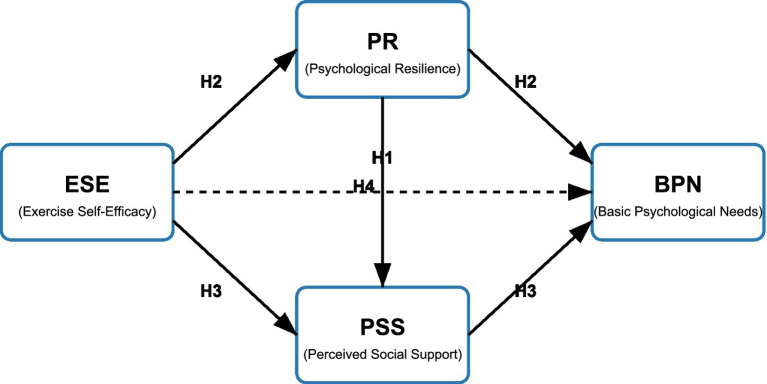
Hypothesized chain mediation model.

In summary, this study aims to explore the mechanisms by which exercise self-efficacy affects basic psychological needs among flight cadets, analyzing the chain mediation effects of psychological resilience and perceived social support. By integrating social cognitive theory, self-determination theory, and related theories of psychological resilience and social support, we construct a more complete chain mediation model (Figure 1), hoping to provide theoretical basis and practical guidance for mental health interventions among flight cadets.

## 2 Participants and methods

### 2.1 Participants

This study employed a cross-sectional design, using cluster sampling to conduct a survey among freshman and sophomore flight cadets at the Civil Aviation Flight University of China. Data was collected through online questionnaires distributed to participating flight cadets, containing measurement items on exercise self-efficacy, basic psychological needs, perceived social support, and psychological resilience. To ensure data quality, researchers screened the collected questionnaires and eliminated those completed in extremely short periods of time. Considering the training model and management characteristics of flight cadets, cluster sampling by class was adopted. The specific procedures were as follows: (1) First, a complete list of all enrolled flight cadet classes was obtained, including all teaching classes from first-year and second-year levels; (2) Simple random sampling was used to randomly select 14 participating classes from the first-year and second-year levels; (3) Questionnaire surveys were conducted on all cadets in the selected classes. At the time of survey (December 24, 2024), the overall distribution of enrolled flight cadets at the Civil Aviation Flight University of China was: 1,487 first-year cadets (56.9%) and 1,126 second-year cadets (43.1%), totaling 2,613 individuals. The final sample in this study included 301 first-year cadets (57.4%) and 223 second-year cadets (42.6%). Chi-square goodness-of-fit test results showed no significant difference between the grade distribution of the research sample and the overall grade distribution (*χ*^2^ = 0.063, df = 1, *p* = 0.802), indicating that the research sample has good representativeness in terms of grade composition. Data were collected through online questionnaires distributed to participating cadets. The questionnaire contained measurement items for exercise self-efficacy, basic psychological needs, perceived social support, and psychological resilience. Inclusion criteria included: (1) Cadets currently enrolled at the Civil Aviation Flight University of China; (2) Voluntary participation in the survey. Exclusion criteria included: (1) Completion time screening—based on the questionnaire length (75 items) and normal reading speeds, we set the minimum reasonable completion time at 300 s (5 min) to prevent speeding and careless responding; (2) Completeness screening—responses with missing data on key variables were excluded. A total of 558 cadets were invited to participate in the survey. After applying these quality control measures, 34 responses were excluded (31 for completion time <300 s, 3 for incomplete data), resulting in 524 valid responses and a final valid response rate of 93.91%. Among the final valid sample (*N* = 524), the majority were male (*n* = 522, 99.6%), with 2 female participants (0.4%). The age of participants ranged from 17 to 23 years (Mean = 19.2, SD = 1.3). Regarding grade level, 301 (57.4%) were freshmen and 223 (42.6%) were sophomores. In terms of family residence, 33.3% came from urban areas and 66.7% from rural areas.

### 2.2 Research instruments

Data collection was administered via an online survey platform (Questionnaire Star). The digital instrument compiled demographic questions and the four validated psychological scales described below.

1. Exercise self-efficacy was measured using the General Self-Efficacy Scale (GSES) developed by Schwarzer et al. (1997) and Tikac et al. (2022). To ensure context specificity, the items were modified to specifically refer to exercise situations. Given that the scale items were adapted to specific exercise scenarios, a content validity assessment was conducted. A panel of three experts (one professor in sport psychology and two doctoral researchers) reviewed the items for relevance and clarity. Experts rated each item on a 4-point scale (1 = not relevant, 4 = highly relevant). All items received ratings of 3 or 4 from the experts, resulting in an Item-Level Content Validity Index (I-CVI) of 1.00. Minor linguistic adjustments were made based on expert feedback to improve clarity. The final adapted items are listed in the Supplementary material. This scale consists of nine items, each describing confidence in persisting with exercise in different situations, using a 10-point scoring method ranging from “No confidence at all” to “Complete confidence.” The scale does not include reverse-scored items; higher scores indicate greater confidence and stronger self-efficacy. In this study, the Cronbach’s *α* coefficient of this scale is 0.978. The Chinese version of GSES has demonstrated good reliability and validity in previous studies with Chinese populations by Wei et al. (2008), with reported Cronbach’s α of 0.853.
2. Perceived social support was measured using the Multidimensional Scale of Perceived Social Support originally developed by Blumenthal et al. (1987), Zimet et al. (1990), and translated into Chinese by Jiang (2001) This scale assesses the participants’ level of perceived social support, comprising three dimensions: family support, friend support, and significant others support, with a total of 12 items. Each item employs a 7-point Likert scale, ranging from 1 to 7, corresponding to “Strongly disagree” to “Strongly agree,” with total scores ranging from 12 to 84 points. Higher scores indicate higher levels of perceived social support. The Cronbach’s *α* coefficient of this scale in this study was 0.981. The Chinese version has shown strong psychometric properties in Chinese samples by Liu et al. (2022), with factor loadings ranging from 0.504 to 0.995 across the three dimensions.
3. Psychological resilience was measured using the Connor-Davidson Resilience Scale (CD-RISC) developed by Connor and Davidson (2003). This scale has been widely applied in psychological resilience research among high-stress occupational groups and has demonstrated good reliability and validity. The scale includes five dimensions: resilience, self-efficacy, positive acceptance of change, sense of control, and spiritual influence, with a total of 25 items. It employs a 5-point Likert scale, ranging from “Not true at all” to “True nearly all the time,” scored from 1 to 5 points, with total scores ranging from 25 to 125. Higher scores indicate higher levels of psychological resilience. The Cronbach’s *α* coefficient of this scale in this study was 0.984. The Chinese version of CD-RISC has been validated in various Chinese populations by Li et al. (2008), showing good reliability (*α* = 0.62–0.85 across 11 factors) and satisfactory factor structure with 55.77% cumulative variance explained.
4. Basic psychological needs were measured using the Basic Psychological Needs Scale (BPNS) developed by Deci and Ryan (2000) and Lourenço et al. (2022) to assess the satisfaction of flight cadets’ basic psychological needs. This scale consists of three dimensions: need for autonomy, need for competence, and need for relatedness, with a total of 21 items. Among these, the need for autonomy dimension includes seven items, mainly measuring the degree of autonomous choice and decision-making regarding one’s behavior; the need for competence dimension includes six items, assessing opportunities to demonstrate and develop abilities in work; the need for relatedness dimension includes eight items, measuring the need to establish close relationships with others. The Cronbach’s *α* coefficient of the scale in this study is 0.783. The Chinese adaptation of BPNS by Liu et al. (2013) has shown adequate reliability across the three dimensions: autonomy (*α* = 0.75), competence (*α* = 0.68), and relatedness (*α* = 0.83) in Chinese samples.

All scales used in this study were previously translated and validated Chinese versions. The GSES and MSPSS have been widely used in Chinese populations with established psychometric properties. For the CD-RISC and BPNS, cultural adaptation involved back-translation procedures and pilot testing with 50 flight cadets to ensure cultural appropriateness and clarity of items. Minor linguistic adjustments were made to three items in the BPNS to better reflect the flight training context while maintaining construct validity.

### 2.3 Research methods

SPSS 26.0 (IBM Corporation, Armonk, New York) and AMOS 28.0 (IBM Corporation, Armonk, New York) were used to process the data, and the validity of the scales was confirmed before use. First, SPSS 26.0 statistical analysis software was used to recode reverse-scored items as the same variables and standardize the data, followed by exploratory factor analysis and confirmatory factor analysis on the processed data. Descriptive statistics required calculating means and standard deviations, and Pearson correlations were used to reveal relationships between variables. Linear regression used exercise self-efficacy as the independent variable, with basic psychological needs, psychological resilience, and perceived social support as dependent variables to determine predictors of the dependent variables. After SPSS analysis, AMOS 28.0 was used for path analysis to examine the relationships to examine the relationships between exercise self-efficacy, basic psychological needs, psychological resilience, and perceived social support.

## 3 Results

### 3.1 Common method bias test

The data in this study were collected through an online self-assessment method (Questionnaire Star platform). Anonymous methods and reverse scoring were used to control measurement bias. Harman’s single factor test was applied to analyze all items involved in this study. Results showed that exploratory factor analysis extracted 6 factors with eigenvalues greater than 1, with the first factor explaining 27.997% of the variance, which is well below the critical value of 40%. This indicates that common method bias did not significantly affect the research data.

### 3.2 Descriptive statistics

Descriptive statistical analysis revealed that flight cadets (*N* = 524) demonstrated moderate to high levels across all measured constructs (Table 1). Exercise self-efficacy scores (M = 58.81, SD = 25.50) indicated moderate levels of exercise confidence, representing 65.3% of the maximum possible score. Basic psychological needs were relatively well satisfied (M = 94.80, SD = 15.75, 64.5% of maximum), with need for relatedness (M = 33.57, SD = 7.10) scoring higher than need for autonomy (M = 32.09, SD = 6.03) and need for competence (M = 29.13, SD = 6.53). This pattern suggests that flight cadets experience stronger interpersonal connections compared to feelings of autonomy and competence in their training environment.

**Table 1:** Descriptive statistics and reliability analysis.

Variable	*N*	Mean	SD
Exercise self-efficacy (ESE)	524	58.81	25.497
Basic psychological needs (BPN)	524	94.80	15.754
Need for autonomy (NA)	524	32.09	6.025
Need for competence (NC)	524	29.13	6.525
Need for relatedness (NR)	524	33.57	7.098
Psychological resilience (PR)	524	90.01	25.085
Resilience (Re)	524	17.69	5.153
Self-efficacy (Se)	524	18.33	5.329
Positive acceptance of change (PAC)	524	18.18	5.465
Sense of control (SC)	524	17.85	5.155
Spiritual influence (SI)	524	17.96	5.237
Perceived social support (PSS)	524	62.48	17.630
Family support (FS)	524	20.89	6.014
Friend support (FrS)	524	20.78	6.072
Significant others support (SOS)	524	20.81	6.023

ESE, exercise self-efficacy; BPN, basic psychological needs; PR, psychological resilience.

Psychological resilience levels were high (M = 90.01, SD = 25.09, 72.0% of maximum), with relatively balanced scores across all five dimensions (range: 17.69–18.33), indicating participants’ adequate capacity for stress management and adaptation. Perceived social support was moderately high (M = 62.48, SD = 17.63, 74.4% of maximum), with similar levels across family support (M = 20.89, SD = 6.01), friend support (M = 20.78, SD = 6.07), and significant others support (M = 20.81, SD = 6.02), reflecting balanced support from multiple sources. The substantial standard deviations across variables (ranging from 15.8 to 43.4% of the mean) indicate considerable individual differences among participants.

### 3.3 Correlation analysis of exercise self-efficacy, basic psychological needs, psychological resilience, and perceived social support among civil aviation flight cadets

**Figure 2:**
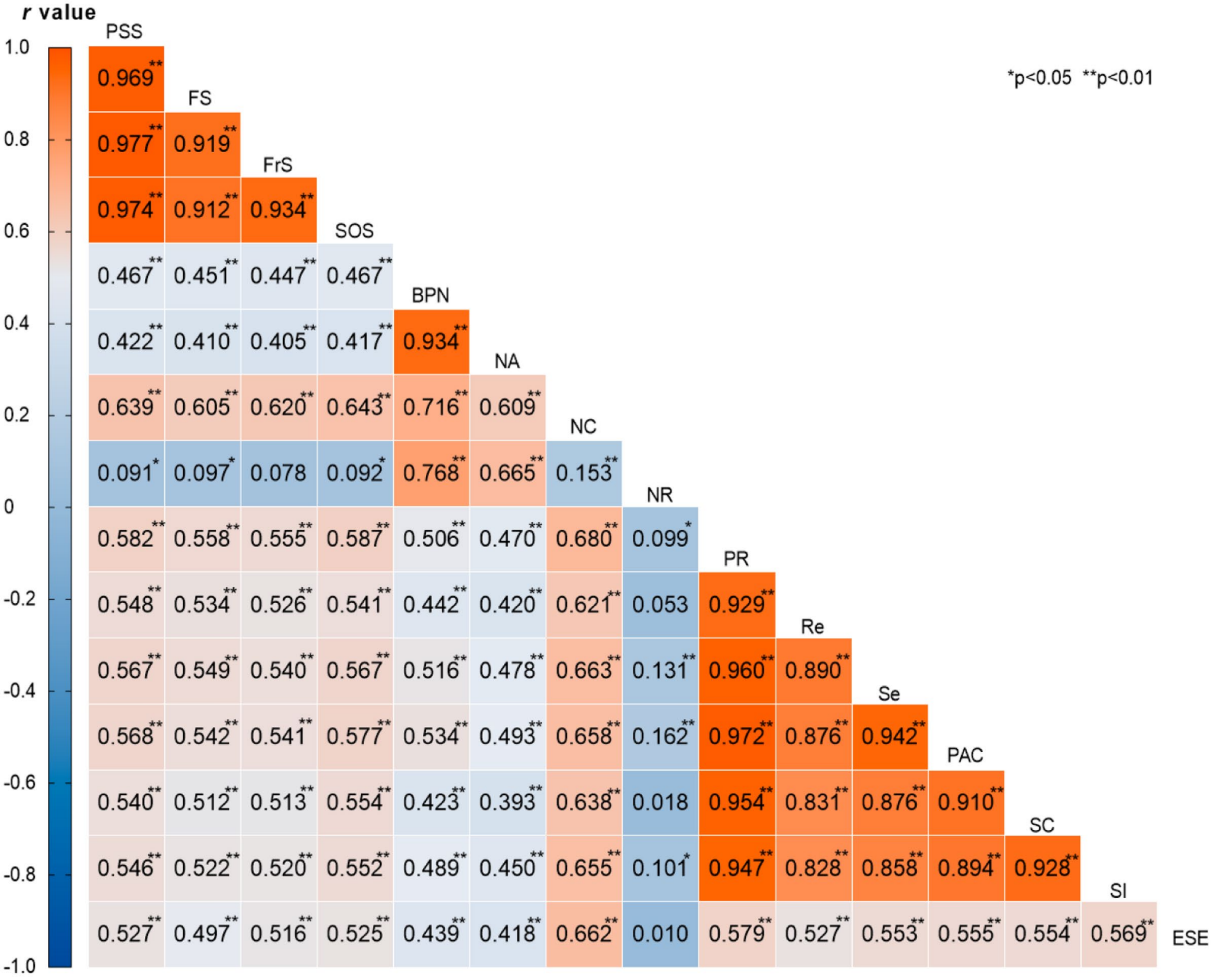
Correlation heatmap of variables and factors. PSS, perceived social support; FS, family support; FrS, friend support; SOS, significant others support; BPN, basic psychological needs; NA, need for autonomy; NC, need for competence; NR, need for relatedness; PR, psychological resilience; Re, resilience; Se, self-efficacy; PAC, positive acceptance of change; SC, sense of control; SI, spiritual influence; ESE, exercise self-efficacy.

Pearson correlation analysis revealed significant positive associations among most study variables (Figure 2). Exercise self-efficacy demonstrated a large positive correlation with total basic psychological needs (*r* = 0.439, *p* < 0.01, Cohen’s *d* = 1.03), indicating that higher exercise confidence is substantially associated with greater psychological need satisfaction. The strongest correlation was observed between exercise self-efficacy and need for competence (*r* = 0.662, *p* < 0.01, Cohen’s *d* = 1.85), representing a very large effect size and suggesting that exercise confidence is particularly linked to feelings of capability and effectiveness.

Notable finding, Exercise self-efficacy showed no significant correlation with need for relatedness (*r* = 0.010, *p* > 0.05, Cohen’s *d* = 0.02), indicating a negligible relationship. This unexpected finding may reflect the unique characteristics of the military-style flight training environment, where interpersonal relationships are largely structured by institutional arrangements rather than individual exercise confidence levels. Exercise self-efficacy showed large positive correlations with psychological resilience (*r* = 0.579, *p* < 0.01, Cohen’s *d* = 1.44) and perceived social support (*r* = 0.527, p < 0.01, Cohen’s *d* = 1.21). The correlation coefficients across resilience dimensions were consistently large (range: *r* = 0.527–0.569, all *p* < 0.01), suggesting that exercise confidence relates to multiple aspects of adaptive capacity. Among mediating variables, psychological resilience and perceived social support showed a large positive correlation (*r* = 0.582, *p* < 0.01, Cohen’s *d* = 1.46), supporting the proposed chain mediation pathway. Need for competence demonstrated particularly strong associations with all resilience dimensions (range: *r* = 0.621–0.680, all *p* < 0.01), indicating that feelings of competence are closely tied to various aspects of psychological resilience. Effect Size Interpretation Following Cohen’s (1988) conventions, correlations were interpreted as small (*r* ≥ 0.10), medium (*r* ≥ 0.30), and large (*r* ≥ 0.50). Most correlations in our study fell into the medium to large range, indicating meaningful associations among constructs.

### 3.4 Measurement model assessment

To ensure the psychometric rigor of the instruments used in this study, a confirmatory factor analysis (CFA) was conducted for the multi-dimensional constructs (Psychological Resilience and Perceived Social Support) using AMOS 28.0. As shown in Table 2, the standardized factor loadings for all items ranged from 0.904 to 0.976, exceeding the recommended threshold of 0.50. The Average Variance Extracted (AVE) values were 0.883 for Psychological Resilience and 0.922 for Perceived Social Support, both surpassing the 0.50 benchmark. Additionally, the Composite Reliability (CR) values were 0.974 and 0.972, respectively, indicating excellent internal consistency reliability.

**Table 2:** Confirmatory factor analysis results for latent variables.

Latent Variable	Item	Factor loading (*λ*)	AVE	CR
PR	Re	0.904	0.883	0.974
	Se	0.956		
	PAC	0.976		
	SC	0.936		
	SI	0.925		
PSS	FrS	0.968	0.922	0.972
	SOS	0.965		
	FS	0.947		

AVE, average variance extracted; CR, composite reliability. All factor loadings are significant at *p* < 0.001. PSS, perceived social support; FS, family support; FrS, friend support; SOS, significant others support; PR, psychological resilience; Re, resilience; Se, self-efficacy; PAC, positive acceptance of change; SC, sense of control; SI, spiritual influence.

### 3.5 Path analysis of the hypothesized chain mediation model

**Figure 3:**
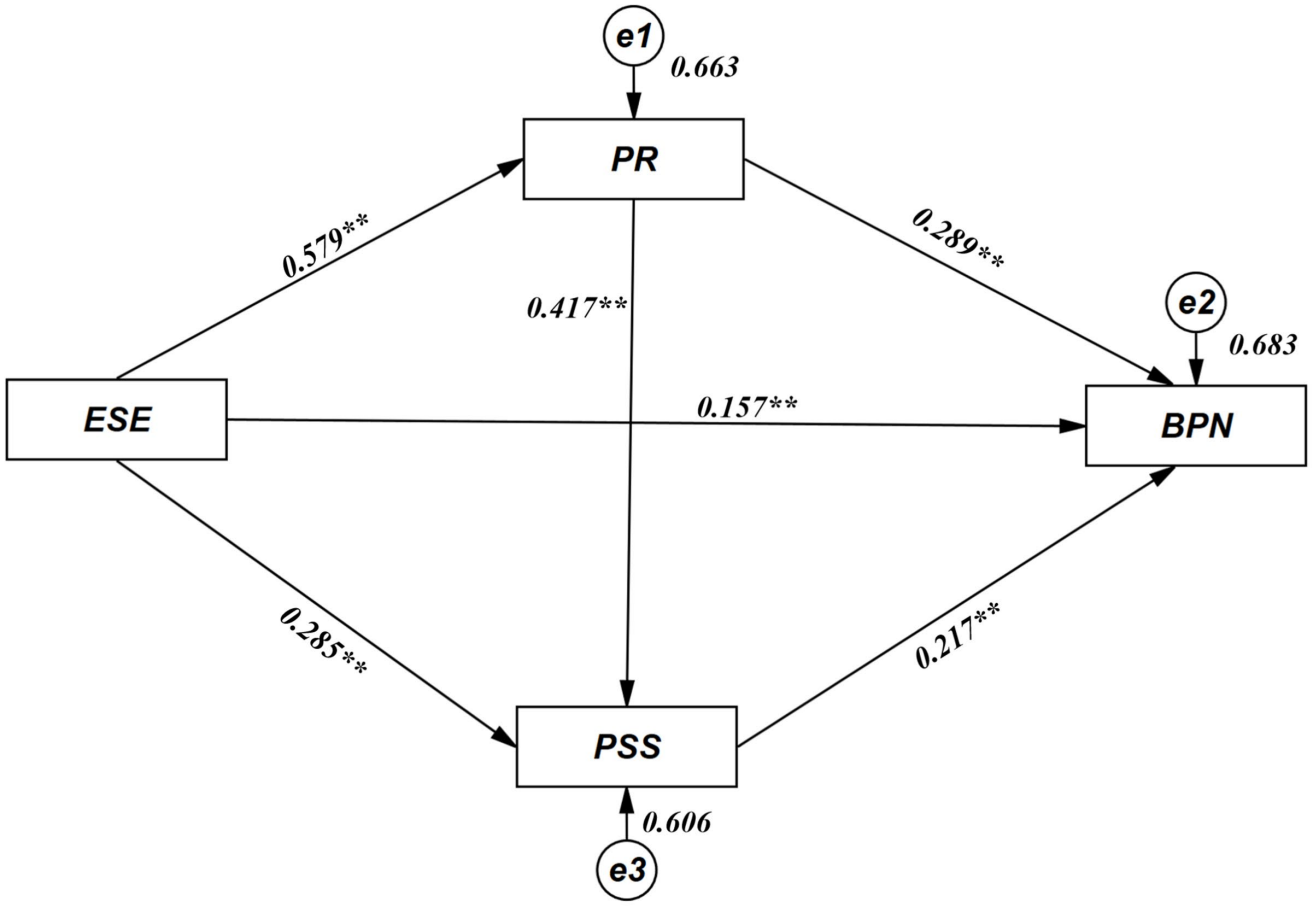
Path analysis results of the hypothesized chain mediation model (standardized estimates).

This study used multiple mediation effect testing procedures, analyzing the influence of exercise self-efficacy on basic psychological needs and the chain mediating role of psychological resilience and perceived social support through Amos 28.0 path model. Control variables included gender, height, weight, age, place of family residence, and grade level. As shown in Figure 3 and Table 3, exercise self-efficacy significantly and positively predicted psychological resilience, indicating that flight cadets with higher exercise confidence also demonstrated higher levels of psychological resilience. Similarly, exercise self-efficacy significantly positively predicted perceived social support. Furthermore, exercise self-efficacy showed significant positive predictive effects on basic psychological needs, particularly on the need for autonomy.

**Table 3:** Mediation model testing (*n* = 524).

Structural path	Unstd. estimate (B)	S.E.	C.R. (t)	Std. estimate (β)	*p*
ESE ⇒ PR	0.579	0.036	16.259	0.579	<0.001
ESE ⇒ PSS	0.285	0.042	6.835	0.286	<0.001
ESE ⇒ BPN	0.157	0.046	3.402	0.157	<0.001
PR ⇒ PSS	0.417	0.042	9.969	0.417	<0.001
PR ⇒ BPN	0.289	0.048	5.964	0.289	<0.001
PSS ⇒ BPN	0.217	0.046	4.668	0.217	<0.001

ESE, exercise self-efficacy; PR, psychological resilience; PSS, perceived social support; BPN, basic psychological needs.

Regarding relationships between mediating variables, psychological resilience significantly positively predicted perceived social support. The mediating variables’ effects on the dependent variable showed that psychological resilience significantly positively predicted basic psychological needs, and perceived social support also significantly positively predicted basic psychological needs.

Among control variables, age negatively predicted basic psychological needs, while place of family residence showed a slight positive predictive effect on basic psychological needs. Other control variables such as gender, height, weight, and grade level had no significant predictive effects on any dependent variables.

In summary, exercise self-efficacy can both directly influence basic psychological needs and indirectly affect basic psychological needs through the chain mediating pathway of psychological resilience and perceived social support. This indicates that enhancing flight cadets’ exercise self-efficacy can not only directly strengthen their basic psychological needs satisfaction but also indirectly promote the fulfillment of basic psychological needs by improving psychological resilience and enhancing perceived social support.

The Bootstrap method (5,000 resamples) was further used to test the mediating effects of psychological resilience and perceived social support between exercise self-efficacy and basic psychological needs. As shown in Table 4, exercise self-efficacy had both significant direct effects on basic psychological needs and significant influence through three indirect pathways. Specifically, the indirect effect through psychological resilience accounted for the largest proportion of the total effect, followed by the indirect effect through perceived social support, and finally the chain mediating effect.

**Table 4:** Summary of mediation effects.

Effect	Path	Effect (Std.)	% of Total effect
Direct effect	ESE ⇒ BPN	0.157	35.8%
Indirect effect	ESE ⇒ PR ⇒ BPN	0.167	38.1%
	ESE ⇒ PSS ⇒ BPN	0.062	14.2%
	ESE ⇒ PR ⇒ PSS ⇒ BPN	0.052	11.9%
Total effect		0.439	100%

## 4 Discussion

### 4.1 The direct relationship between exercise self-efficacy and basic psychological needs in flight cadets

This study found that exercise self-efficacy was significantly positively correlated with the total score of flight cadets’ basic psychological needs (*β* = 0.439, *p* < 0.001), indicating a strong association between exercise self-efficacy levels and the satisfaction of basic psychological needs among flight cadets. This result supports the hypothesis of a positive association between exercise self-efficacy and the basic psychological needs status of flight cadets. However, it should be noted that due to the cross-sectional design of this study, this result can only demonstrate a statistical association between the two variables, rather than a definitive causal relationship. This result can be explained from two perspectives. First, flight cadets with high exercise self-efficacy may simultaneously possess stronger self-regulation abilities and positive emotional experiences (Zhao et al., 2023). According to self-determination theory, when individuals’ needs for autonomy, competence, and relatedness are satisfied, they experience higher levels of psychological well-being (Sheldon, 2011). Data analysis showed that exercise self-efficacy had the strongest correlation with competence needs (*r* = 0.662, *p* < 0.01), which may indicate a close association between positive evaluation of one’s physical fitness and confidence in facing challenges, potentially influencing the satisfaction of competence needs. This finding is consistent with research by Huang et al. (2025), who found that exercise self-efficacy may be an important protective factor for promoting individual psychological health, especially for groups in high-stress environments. Bandura’s (1977) social cognitive theory also points out that self-efficacy can shape individuals’ thinking patterns and emotional responses, thereby influencing their physiological state and behavioral performance (Locke, 1997). Second, flight cadets with higher levels of exercise self-efficacy are typically associated with healthy and positive lifestyle orientations. According to social cognitive theory, high self-efficacy beliefs often act as a cognitive resource that motivates individuals to adopt active coping strategies (Wang et al., 2022). They tend to actively engage in social interactions and cultivate diverse interests, which may promote the satisfaction of autonomy needs (*β* = 0.157, *p* < 0.01). Research data indicate that exercise self-efficacy has a significant direct effect on basic psychological needs (direct effect = 0.157), accounting for 35.8% of the total effect, highlighting the important direct role of exercise self-efficacy. The training environment for flight cadets has unique characteristics, including high-intensity physical requirements, strict disciplinary constraints, and elimination pressure. In this context, the positive role of exercise self-efficacy is particularly prominent. Exercise self-efficacy, as a psychological resource, not only strengthens flight cadets’ belief in their ability to manage stress but may also enhance their confidence and tolerance when facing challenges and tolerance when facing challenges, thereby reducing the negative impact of training stress on basic psychological needs satisfaction (Tyne et al., 2024). Specifically, exercise self-efficacy may function as a psychophysiological “dampening” mechanism. By strengthening the belief in one’s physical capabilities, it helps flight cadets maintain optimal physiological arousal levels during strenuous physical training, preventing the performance deterioration often caused by anxiety-induced physiological hyperarousal. Greenleaf et al. (2010) found through research on high-stress occupational groups that exercise self-efficacy is an important mediating variable that regulates the relationship between occupational stress and psychological health. Correlation analysis shows that exercise self-efficacy is moderately positively correlated with various dimensions of psychological resilience (resilience, self-efficacy, positive acceptance of change, sense of control, spiritual influence) (*r* = 0.527–0.569, *p* < 0.01), indicating that exercise self-efficacy can promote the development of multiple psychological qualities in flight cadets.

It is noteworthy that this study found no significant correlation between exercise self-efficacy and relatedness needs (*r* = 0.010, *p* > 0.05), which differs from previous research results on general college student populations and requires in-depth analysis from multiple levels. First, from a cultural background perspective, Chinese collectivist culture emphasizes group harmony and collective identity (Zhou et al., 2023), Flight cadets’ relatedness needs may be more reflected in the pursuit of team belonging and collective honor, while the association with individual exercise performance may be relatively weak. Second, from the perspective of situational specificity, flight cadets are in a highly structured military management environment where their interpersonal networks are relatively fixed and strictly controlled, and social interaction patterns are relatively stable, possibly not easily influenced by individual exercise self-efficacy levels. Research by Bekesiene (2023) further confirms that in closed military academy environments, the impact of personal self-efficacy on interpersonal relationships is significantly weaker than in open environments. Additionally, from a theoretical perspective, according to self-determination theory, the core of relatedness needs is belonging, intimacy, and being cared for. These deep psychological needs are often satisfied through emotional support, mutual understanding, and long-term companionship, rather than short-term ability demonstration or achievement acquisition (Tao and Yu, 2025). This unexpected finding reminds us that in specific populations, the associations between variables may present patterns different from general theoretical expectations. This difference may reflect the uniqueness of the flight cadet population and may also suggest limitations in the applicability of existing theories to special occupational groups. Furthermore, the study found that age had a significant negative predictive effect on basic psychological needs (*β* = −0.148, *p* < 0.001; *β* = −0.118, *p* < 0.01), indicating that as age increases, flight cadets may face greater training pressure and career crises, leading to decreased satisfaction of basic psychological needs. This unexpected finding may reflect the limitations of this study’s sample composition, which only included first and second-year cadets. The age effect may actually reflect the increasing training intensity and elimination pressure brought by academic progression, rather than a true age maturation effect. Additionally, this result contradicts previous research findings that age growth is usually accompanied by improved psychological maturity (Roberts et al., 2006), suggesting that in high-pressure selection environments, the traditional age-psychological development relationship may not apply. Future research should expand the age range and introduce longitudinal designs to verify the stability and causal mechanisms of this effect. In summary, exercise self-efficacy may promote the satisfaction of flight cadets’ basic psychological needs through both direct and indirect pathways. Based on this finding, civil aviation flight academies can improve flight cadets’ exercise self-efficacy levels by scientifically designing physical training programs, conducting targeted confidence-building activities, and implementing physical achievement incentive mechanisms, thereby promoting the satisfaction of their basic psychological needs, maintaining psychological health, and improving training quality and effectiveness. At the same time, attention should be paid to senior and older flight cadets, providing them with more targeted psychological support and self-efficacy enhancement strategies to help them better cope with challenges in their career development process.

### 4.2 The mediating role of psychological resilience

Data analysis indicates that exercise self-efficacy may indirectly associate with the satisfaction of flight cadets’ basic psychological needs by improving their psychological resilience levels, verifying hypothesis H2, with the mediating effect accounting for 38.1% of the total effect. It should be noted that previous research has mostly focused on general college student populations (Navickienė and Vasiliauskas, 2024; Liu and Huang, 2021), while relatively neglecting the special occupational pressures faced by flight cadets, such as stricter selection standards and elimination systems. This unique background may make the association between exercise self-efficacy and psychological resilience more prominent in this population. Social Cognitive Theory (SCT) focuses on the dynamic interaction between individual cognition, environment, and behavior, providing a theoretical foundation for explaining how self-efficacy influences psychological needs by shaping positive adaptive behaviors (Riley et al., 2016).

First, cadets with high self-efficacy may hold positive beliefs about their abilities. This optimistic self-evaluation may prompt them to view stressful situations as “challenges” rather than “threats,” thereby adopting more resilient and flexible coping strategies, which may be associated with improved psychological resilience levels (Bandura, 1977). A recent study of 251 military pilots found that self-efficacy significantly affected pilots’ ability to handle special situations through the mediating role of psychological resilience, validating the core role of self-efficacy in aviation psychology. The study particularly pointed out that pilots with high self-efficacy could improve their psychological resilience, thereby enhancing their ability to handle special situations (Qiu et al., 2023). Furthermore, existing research indicates that military trauma survivors with high self-efficacy tend to actively summarize lessons learned and adjust their mindset, rather than passively avoiding, which may be a concentrated manifestation of the “resilience” and “positive acceptance of change” dimensions of psychological resilience (Morison and Benight, 2022).

Second, social cognitive theory emphasizes the shaping effect of environmental factors on individual behavior (Cooper et al., 2020). In the training process of flight cadets, environmental factors such as instructor demonstrations and training atmosphere may affect their psychological resilience levels by influencing cadets’ self-efficacy. As emphasized by Gupta and McCarthy (2022), the formation of psychological resilience is rooted in the interactive process between individuals and their environment. On one hand, when instructors demonstrate good coping methods, cadets may more quickly master corresponding cognitive strategies and behavioral skills through observational learning, enhancing self-efficacy. On the other hand, when teams form a collective culture of mutual assistance and friendship, cadets may continuously strengthen their self-efficacy through peer interactions and subsequently adopt more positive and optimistic coping methods. This finding theoretically enriches the application of social cognitive theory in special occupational groups and highlights the unique value of self-efficacy. However, this finding should be interpreted cautiously. Although statistical analysis supports the mediating effect of psychological resilience, the cross-sectional design limits our inference about the temporal sequence relationships between variables (Toh and Hernán, 2008; Savitz and Wellenius, 2023). Additionally, psychological resilience, as a complex psychological construct, may have its association with exercise self-efficacy moderated by multiple factors, including individual differences, training stages, environmental support, etc. Future research needs to adopt longitudinal designs to verify the stability and directionality of this association.

### 4.3 The mediating role of perceived social support

Data analysis shows that exercise self-efficacy promotes the satisfaction of basic psychological needs by enhancing perceived social support, verifying hypothesis H3, accounting for 14.2% of the total effect. Bioecological theory emphasizes that individual development is embedded in multi-level social ecological systems, and interactions between different system elements may shape individual behavior (Tong and An, 2024). Cadets with higher exercise self-efficacy may exhibit stronger interpersonal tendencies, such as better listening and expression abilities, which may help them establish effective support networks (Peng et al., 2025). At the interpersonal level, positive family relationships and peer interactions may directly affect cadets’ subjective perception of support (An et al., 2024). At the institutional level, although military-style management may objectively limit cadets’ access to external support channels, it may also prompt them to more cherish and utilize internal support resources (Chiu et al., 2024). A qualitative study involving pilots from nine civil aviation companies found that despite companies establishing psychological counseling rooms, most pilots were still unwilling to use these services, fearing negative impacts on their future career development. Social support was proven to be an effective solution for improving pilot psychological health (Cahill et al., 2023). Further analysis found that perceived social support is an important bridge connecting multiple system levels. On one hand, good self-efficacy may help cadets establish close connections with classmates and teachers, making them perceive more support. On the other hand, this support perception may strengthen cadets’ self-efficacy (Wang et al., 2024), forming a positive cycle. Therefore, individual factors and environmental elements are integrated through the mediating role of perceived social support, jointly affecting cadets’ psychological adaptation outcomes. This indicates that positive interaction between individuals and environment may be an important pathway for cadets to obtain psychological energy. However, in the special environment of military-style management, perceived social support may also present a “double-edged sword” effect. On one hand, moderate social support helps alleviate training pressure and promote psychological adaptation; on the other hand, excessive dependence on external support may weaken cadets’ autonomous development abilities. This reminds us to formulate policies according to local conditions and construct rich and complementary social support networks for cadets.

### 4.4 The chain mediating effect of psychological resilience and perceived social support

Chain mediation testing further verified hypothesis H4, indicating that exercise self-efficacy may affect the satisfaction of basic psychological needs through the chain association of psychological resilience and perceived social support. This finding suggests that there may be positive interactions between individual psychological capital accumulation and social capital acquisition, jointly promoting the healthy development of civil aviation flight cadets. Positive organizational behavior provides an explanatory perspective for the establishment of hypothesis H4. Exercise self-efficacy, as a precursor to psychological capital, may provide an important foundation for cadets to accumulate psychological resilience. Cadets with high self-efficacy may exhibit more optimistic and positive emotional experiences and cognitive patterns, which not only may enhance their courage to face setbacks but also improve their confidence in problem-solving, thereby forming good psychological capital reserves (Khan et al., 2024). Carter and Youssef-Morgan (2022) found that psychological capital (including self-efficacy, optimism, hope, resilience) is positively correlated with individuals’ stress coping methods, well-being, and work performance. Individuals with high psychological capital may adopt more positive coping strategies when facing stress, such as positive reappraisal and seeking help, while less frequently using negative strategies such as avoidance.

Although direct validation studies of the complete chain pathway are still limited, previous research provides valuable references for understanding the associations between various links. Lin et al. (2025) found through a survey of 898 college students that physical exercise significantly affected psychological capital including self-efficacy, resilience, hope, and optimism through the chain mediating role of perceived social support and self-control, providing direct evidence for physical activities affecting psychological capital through social psychological mechanisms. Furthermore, Zhang et al. (2024) showed through research on 1,379 vocational college students that self-efficacy and resilience have significant mediating effects in the relationship between social support and psychological health outcomes. Although the variable sequence in that study differs from the hypothesis of this study, it verified the close association between self-efficacy, resilience, and social support. Meanwhile, in research on 400 Chinese students, researchers found that the relationship between physical activity and psychological health was completely realized through the chain mediating effect of self-efficacy and stress self-management, further confirming that sports-related self-efficacy can affect psychological health outcomes through psychological resilience-related factors (Zhang et al., 2024). It should be emphasized that although these studies support the associations between related variables, they differ from this study in aspects such as variable operationalization definitions, research population characteristics, and pathway directions. The value of this study lies in first verifying the complete chain pathway of “exercise self-efficacy → psychological resilience → perceived social support → basic psychological needs” in the special population of flight cadets, filling the gap in existing research regarding direct validation of this theoretical sequence.

### 4.5 Research limitations and future directions

This study, from the perspective of flight cadets, deeply explored the mechanisms by which exercise self-efficacy affects basic psychological needs through psychological resilience and perceived social support, enriching the theoretical connotations of social cognitive theory, bioecological theory, and positive organizational behavior. It has important significance for guiding flight cadets in improving psychological adaptation abilities and satisfying basic psychological needs. The research results indicate that psychological resilience and perceived social support play chain mediating roles between exercise self-efficacy and basic psychological needs, providing valuable references for constructing psychological health development models for flight cadets. Despite these findings, this study still has some limitations. First, regarding research design, this study adopted a cross-sectional design that cannot clarify causal relationships between variables. Future research should adopt longitudinal tracking designs, with recommendations for at least 2 years of tracking surveys of flight cadets, measuring each variable every 6 months to verify the temporal sequence causal effects of exercise self-efficacy, psychological resilience, and perceived social support on basic psychological needs. Quasi-experimental research can also be designed, using randomized controlled trials to verify the effects of resilience training interventions, with experimental groups receiving 12 weeks of structured resilience training and control groups maintaining routine training, comparing changes in variables before and after intervention. Second, regarding measurement methods, multi-source data collection strategies should be adopted to reduce common method bias, including combining objective measurement methods such as instructor evaluations, peer nominations, and physiological indicator monitoring (such as cortisol levels, heart rate variability) to construct multi-dimensional assessment systems. Third, regarding sample expansion, future research should include flight cadet samples from different types of aviation academies, comparing differences between military and civil aviation training environments, while conducting cross-cultural research to verify the model’s applicability in different cultural backgrounds. Finally, regarding theoretical deepening, it is recommended to integrate positive psychology theory and stress coping theory, explore more complex moderating variables and mediating pathways such as training stages and personal traits, and develop standardized psychological resilience training programs specifically for flight cadets to provide evidence-based practice guidance for aviation education and training. Fourth, while procedural remedies and Harman’s single-factor test were employed, recent research indicates that Harman’s test may not reliably detect common method bias (Howard et al., 2024). Consequently, these results should be interpreted with caution. Future studies are encouraged to adopt more rigorous techniques, such as the CFA marker analysis.

## 5 Conclusion

1. Exercise self-efficacy has a significant positive direct effect on basic psychological needs, with the most prominent impact on competence needs. Accordingly, flight academies should establish personalized physical training goal-setting systems, breaking down physical fitness tests into phased achievement goals, and gradually improving cadets’ exercise self-efficacy through successful experiences. They should also implement peer mutual assistance training mechanisms, arranging outstanding senior cadets as physical training mentors to strengthen junior cadets’ exercise confidence through vicarious experiences.
2. Psychological resilience plays an important mediating role between exercise self-efficacy and basic psychological needs, verifying the key function of psychological resilience as a “catalyst.” Therefore, it is recommended to implement systematic resilience training programs: conduct stress inoculation training, simulating high-pressure situations in flight training such as physical fitness tests under adverse weather conditions; provide cognitive restructuring training, teaching cadets to reframe training setbacks as growth opportunities; establish dynamic resilience monitoring systems, regularly using the CD-RISC scale to track cadets’ psychological resilience development trajectories.
3. Perceived social support exhibits a mediating effect between exercise self-efficacy and basic psychological needs. Based on this finding, a multi-dimensional support network of “instructor-cadet-peer-family” should be constructed, establishing regular teacher-student symposium systems and family open day activities; building cadet mutual assistance record systems; and setting up psychological health liaison positions, with outstanding senior cadets providing peer psychological support.
4. Psychological resilience and perceived social support form a chain mediating effect, reflecting the synergistic action of individual psychological capital and social capital. From this discovery, resilience cultivation should be integrated with support network construction. In response to the negative age effect found in the study, specialized career development counseling and psychological support services should be provided for senior cadets, and intervention effectiveness evaluation mechanisms should be established, regularly using relevant scales to monitor intervention efficacy.

## Data Availability

The original contributions presented in the study are included in the article/Supplementary material, further inquiries can be directed to the corresponding author.
