# Supplementary material for: The effect of exercise self-efficacy on basic psychological needs in flight cadets: the chain mediating role of psychological resilience and perceived social support
Source: Front Psychol. 2026 Jan 14;16:1701055. doi: 10.3389/fpsyg.2025.1701055 (PMC12847292; doi:10.3389/fpsyg.2025.1701055)
Supplement: Supplementary file 1 [file Table_1.DOCX]

**Table S1. Items of the Adapted Exercise Self-Efficacy Scale**

Instruction: Please indicate how confident you are that you can exercise under the following conditions. “0” indicates no confidence at all, and “10” indicates complete confidence.

| **Item No.** | **Original Item (Chinese)** | **Translated Item (English)** |
| --- | --- | --- |
| 1 | 天气使您感到困扰的时候 | When the weather is bothersome |
| 2 | 您对该运动没有兴趣的时候 | When you have no interest in the activity |
| 3 | 运动会感觉疼痛的时候 | When you feel pain or discomfort during exercise |
| 4 | 您一个人运动，没有人作伴的时候 | When you have to exercise alone without a partner |
| 5 | 您觉得没有感受到运动乐趣的时候 | When you do not feel the fun of exercise |
| 6 | 您忙于其他事情的时候 | When you are busy with other matters |
| 7 | 您觉得疲倦的时候 | When you feel tired |
| 8 | 您觉得有压力的时候 | When you feel stressed |
| 9 | 您觉得心情糟糕的时候 | When you are in a bad mood |
